# Relationship between Experience of Client Violence and Turnover Intention among Workers in Long-Term Care Facilities for Older Adults: Focusing on Nurses, Social Workers, and Care Workers

**DOI:** 10.3390/nursrep13010050

**Published:** 2023-03-20

**Authors:** Seunghoon Choi, Hyeri Shin, Minah Lee, Kimyung Han

**Affiliations:** 1 Graduate School of Social Welfare, Yonsei University, Seoul 03722, Republic of Korea; cyoon@yonsei.ac.kr; 2 Department of Gerontology, Graduate School of East-West Medical Science, Kyung Hee University, Yongin 17104, Republic of Korea; zisoa@khu.ac.kr; 3 Department of Policy Research, Korea Disabled People’s Development Institute, Seoul 07236, Republic of Korea; sw.kimyunghan@koddi.or.kr

**Keywords:** violence experience, turnover intention, nurse, social worker, care worker, Korea

## Abstract

To prevent discontinuity of long-term care service and guarantee the quality of care, it is important to clarify the determinants of turnover intentions of long-term care workers. They are at a higher risk of experiencing violence—including physical, emotional, and sexual—from patients or their families, possibly leading to high turnover intention. This study aims to verify how having experienced client violence affect turnover intention of long-term care workers and to suggest implications to prevent frequent turnover in long-term care field. Logistic regression analysis was conducted between groups who have experienced client violence and who have not, using 2019 Korean LTC Survey data. Results revealed that, first, there were differences in determinants of turnover intention depending on groups. Second, having experienced client violence had a different effect on turnover intention based on personal characteristics. Third, gender and occupational differences were found. Based on our results, we highlighted the need for discussions on interventions to address client violence exposure among long-term care workers.

## 1. Introduction

Since its establishment in 2008, Korea’s Long-Term Care Insurance (hereafter LTCI) has functioned as the primary care program for older adults, reducing families’ care burden and guaranteeing recipients’ choice of where to stay. As the LTCI is a social insurance scheme with the same coverage as that of the National Health Insurance, it was able to cover entire nationwide population from the start. As of late 2021, 10.1% of the Korean population over 65 was benefiting from one or more LTCI programs [1].

To meet the growing needs of long-term care (hereafter LTC), a significant number of agencies and workers are required. “LTC workers” include various types of licensed workers, such as registered nurses, nurse aides, care workers, social workers, and occupational and physical therapists, all of whom provide LTC services in home care and institutional settings. While 89.8% of LTC workers are care workers, social workers and nurses/nurse aids comprise only 6.0% and 3.1%, respectively [1]. 

Despite the growing demand for LTC workers, LTC is still not considered to be decent employment, partly due to the low salary, work intensity, and lack of promising career paths to encourage workers to serve for long periods of time [2,3]. LTC workers are notorious for not wanting to work for long periods in the health sector, reporting an average of just three years in Korea [4].

LTC workers also are, similarly to other workers in any social work or hospital field, exposed to client violence. Client violence—mistreatment or abuse experienced in one’s own workplace—is defined as various types of sexual, physical, verbal, and psychological violence or threats from clients toward care workers during the provision of care services [5,6]. 

Previous studies revealed that predictors of client violence in the LTC setting can be observed across three dimensions. First, organizational factors, such as the size of an institution, overtime work, and lack of an organizational risk management system, have been shown to be predictors of violent behaviors perpetrated by LTC recipients, although many studies have focused on cases of care workers [7,8,9]. Second, studies are mostly focused on how and when clients behave violently. LTC workers reported that they were likely to experience client violence while providing daily assistive services, such as changing body position, lifting or moving the client, continence care, changing clothes, and washing [8,10]. Third, individual factors that describe the characteristics of clients, including gender, cognitive function, and ability to perform activities or instrumental activities of daily living (ADL/IADL), were investigated [8,11].

Client violence is not only a threat to LTC workers’ safety but also a risk factor for burnout and low job satisfaction [7,12,13,14,15,16], potentially leading to workers quitting their jobs [6]. Job satisfaction is defined by employees’ attitude toward their jobs based on their work environments [17]. Several researchers found the importance of job satisfaction as the mediator between job-related factors and turnover intentions [18,19]. Turnover intentions reflect workers’ intentions to stay or leave an organization [20], and it is becoming a major global concern, as it could lead to a shortfall in the care workforce in the near future [14,21]. Most of the member countries of the Organisation for Economic Co-operation and Development (OECD) will need additional LTC workers by 2040, with South Korea requiring the most.

Considering the high costs generated by turnover and the extent of poor service quality [21], improvement to the LTC working environment in order to decrease turnover is a key issue. Therefore, identifying the factors related to worker turnover intention and trying to decrease the rate of turnover is significant to an organization’s success [22]. Additionally, from the perspective of clients, frequent turnover impedes the continuity of service and quality of care [23], ultimately decreasing their quality of life.

Previous studies have revealed predictors of turnover intention related to work and job in LTC settings. They include low job satisfaction [24,25,26,27], income [24], work type [24], and job stress [18,24]. The characteristics of care for older adults are also stressors, as they are likely to be medically sophisticated, resulting in an increase in the workload for workers [18]. Traumatic events have a significant relationship with nurses’ turnover intention in intensive care units [28]. Several individual factors have also been found to be related to turnover intention in LTC settings, such as gender [29], age [24], and education [25].

Despite its importance to prevent the discontinuity of LTC service and guarantee the quality of care, studies on the relationship between client violence and the turnover intention of LTC workers in Korea are not yet extensive. Thus, this study aims to verify how exposure to client violence may determine the turnover intention of LTC workers as it is essential to understand the actual situation in the elderly care field to suggest our analysis results as a basic material to use in developing interventions. To achieve the aim of this study, data from the 2019 LTC Survey conducted by the Ministry of Health and Welfare and the Korea Institute for Health and Social Affairs were used to explore the different factors affecting turnover intentions between groups that have and have not experienced client violence.

The research hypothesis and research model for this purpose are as follows.

**Hypothesis** **1.** *The personal and job-related factors of LTC workers will have a significant effect on turnover intention.*

**Hypothesis** **2.** *The personal and job-related factors of LTC workers who experienced client violence will have a significant effect on turnover intention.*

**Hypothesis** **3.** *The personal and job-related factors of LTC workers who did not experienced client violence will have a significant effect on turnover intention.*

## 2. Materials and Methods

### 2.1. Sample and Data

The Long-Term Care Survey was established to meet the demand for long-term care services in South Korea. These are Nationally Approved Statistics (No. 117104) conducted by the Ministry of Health and Welfare and the Statistics Korea based on Article 6-2 (Fact-Finding Survey) of the Long-Term Care Insurance Act. The survey has been conducted every three years since 2019 and uses a stratified systematic sampling method to select approximately 11,000 long-term care beneficiaries, facilities, and staff members nationwide for interviews or internet surveys, using data from the National Health Insurance Corporation’s database. The survey content used for LTC workers in this study includes working patterns and conditions by job type, the type of benefits, experiences of unfair treatment, experiences of disease and accidents, and the improvement of treatment [30].

### 2.2. Measurements

#### Variables in the Model

In this study, personal and job-related factors, which have been shown to affect turnover intention in previous studies, were set as independent variables. The dependent variable was defined as turnover intention, as measured by a question asking whether the respondent planned to change jobs in the future, with a “Yes” response indicating turnover intention. A detailed explanation of the variable configuration in this study can be found in Table 1.

### 2.3. Data Analysis

Stata version 14 was used to determine the descriptive statistics and conduct logistic regression analysis. This study presents the general characteristics, experience of violence, and turnover intentions of nurses, social workers, and care workers. It also conducts an experimental analysis for a deeper understanding of issues. Two models were used to investigate the extent to which client-perpetrated violence affects the turnover intention of LTC workers. According to Model 1, client-perpetrated violence experienced by LTC workers will affect their turnover intention. According to Model 2, the presence or absence of client-perpetrated violence experienced by LTC workers will affect the differences in their turnover intention.

## 3. Results

### 3.1. Descriptive Statistics

Table 2 shows the characteristics of the entire sample. A total of 3541 LTC workers participated in the study. The socio-demographic characteristics of the participants are shown in Table 2.

#### 3.1.1. Gender, Age, and Education Level

In terms of individual factors, the study revealed the following: the proportion of female respondents (91.4%) was higher than that of males and remained high regardless of turnover intention. According to age group, 39.9% of the total respondents were workers in the 50–59 age group. In terms of education level, 42.1% of workers graduated from high school.

#### 3.1.2. Violence Experienced and Turnover Intention

Of the survey respondents, 35.4% reported experiencing violence from clients. Among the types of violence experienced, verbal and psychological violence were the most common (29.7%), followed by physical violence (22.5%), and sexual violence (11.3%). The proportion those who responded that they had an “turnover intention”, namely 17.8% of the total sample, was higher among those who had experienced violence from clients (24.4%) compared to those who had not experienced violence (14.2%).

### 3.2. Descriptive Statistics: Violence Experienced and Turnover Intention

#### 3.2.1. Gender

Table 3 shows the turnover intention according to the characteristics of the group who experienced violence from clients. Firstly, the turnover intention among women who experienced violence from clients was 25%, which was higher than that of men (17%).

#### 3.2.2. Age

By age group, those who experienced violence in the 40–49 age group had the highest turnover intention at 40.5%, followed by those in the 30–39 age group (35.1%) and the 50–59 age group (23.1%).

#### 3.2.3. Type of Occupation

Among LTC workers who experienced violence from clients, social workers showed a higher turnover intention at 37.3% and nurses at 35.7%, while care workers showed a slightly lower intention at 17.8%.

### 3.3. Descriptive Statistics: Comparison of Turnover Intention

Table 4 shows the characteristics of the entire sample with and without turnover intention.

#### 3.3.1. Job and Income Satisfaction

Firstly, the group that is not satisfied with their job showed a higher turnover intention compared to the satisfied group. Turnover intention in the dissatisfied group was between 40% and 41%. It can be seen that turnover intention increases as job satisfaction decreases. Similarly, dissatisfaction with current income also resulted in a higher turnover intention. Specifically, when satisfied with current income, turnover intention was between 4% and 9.7%, while dissatisfaction with current income resulted in a turnover intention of 28.5% to 34.1%.

#### 3.3.2. Type of Employment and Occupation

When comparing job turnover intentions according to employment type, the turnover rate for non-regular workers was 17.8%, which was not significantly different from that of regular workers at 17.9%. On the other hand, among occupations, the percentages of LTC workers with turnover intentions were 28.2% for social workers, 26.6% for nurses, and slightly lower for caregivers at 13.4%.

#### 3.3.3. LTC Benefits and Position

The turnover intention in terms of institutional care benefits for workers was slightly higher at 18.5% than that of benefits for home care service worker at 17.4%. In terms of job position, the turnover intention was highest among assistant and middle managers, at 22.8% and 20.6%, respectively, while facility and office managers had the lowest turnover intention at 5.1% and 15.5%, respectively.

#### 3.3.4. Experience of Accidents and Illnesses Related to Work and Stress Level

The group that experienced accidents or illnesses while working had a higher turnover intentions than the group that did not, and the turnover intention increased with the number of accident or illness experienced. It was also found that the higher the level of stress, the higher the turnover intention.

### 3.4. Relationship between Client Violence and Turnover Intention

#### 3.4.1. Job & Income Satisfaction, Type of Employment

Through logistic regression analysis, we identified the factors affecting the intention to turnover among LTC workers, and the results are presented in Table 5. In preparation for multiple regression analysis, the results of the correlation between the independent variables used in this study revealed no risk of multicollinearity with a correlation of 0.7 or less.

The results show that overall, when job and salary satisfaction were low, the probability of turnover intention increased. Additionally, the probability of turnover intention was higher for those in unstable employment and those in lower positions. This suggests that workers’ turnover intentions involved uncertainty about the future.

#### 3.4.2. Type of Occupation 

The professions that provide services to clients in LTC facilities for older adults can be primarily divided into nurses, social workers, and care workers. Between nurses and care workers, the probability of turnover intention was higher for nurses. 

#### 3.4.3. Experience of Client Violence

Workers who had experienced client violence or accidents and illnesses in their jobs were more likely to have turnover intentions. However, turnover intention based on the type of salary was not found to be significant. 

#### 3.4.4. Age, Gender, Education & Stress Level

The probability of turnover intention was found to be higher among individuals of lower age and higher among women than men. It was also found to be higher among individuals with higher levels of stress and education.

#### 3.4.5. Group That Experienced Client Violence

The next step was to compare turnover intention between groups that did and did not experience client violence; the results are presented in Table 6.

In the case of the group that experienced violence from clients, overall job satisfaction; satisfaction with salary level, occupation, and position; as well as stress were found to be significant factors influencing turnover intention. On the other hand, position and type of salary were found to be insignificant. Factors such as gender, age, and education level have been shown to exert a significant impact on an individual’s turnover intention. Specifically, it was found that individuals who were dissatisfied with their job and salary level had a higher probability of turnover intention. A comparison across occupations reveals that nurses had higher turnover intentions than care workers, and individuals with higher positions and of a higher age had lower turnover intentions. Additionally, the higher the stress and education levels of the respondents, the higher the probability of turnover intention.

#### 3.4.6. Group That Did Not Experience Client Violence

For the group that did not experience client violence, work factors such as overall job satisfaction, income satisfaction, job type, position, and level of stress were found to have a significant effect on turnover intention. On the other hand, position and salary type were found to be insignificant. Among individual factors, age had a significant effect on turnover intention, while gender and education had no significant effect. Specifically, the higher the dissatisfaction with the job and income level, the higher the probability of turnover intention; moreover, the lower the position and age, the higher the probability of turnover intention. In terms of the stress level, the higher the stress level, the higher the probability of turnover intention. With regard to comparisons between occupations, the turnover intention of nurses was found to be higher than that of care workers.

#### 3.4.7. Comparison Analysis

Among the job factors, overall job satisfaction and income satisfaction were found to have a significant effect on turnover intention in both the groups that did and did not experience violence. Specifically, the higher the satisfaction with the income level or the lower the overall job satisfaction, the higher the probability of turnover intention. However, although the income level and job satisfaction of the group who experienced violence was lower than in those who had not, the probability of turnover intention was higher. As for income-level satisfaction, the group that experienced violence had an odds ratio (OR) of 0.58, while the group that did not experience violence had an OR of 0.55, indicating that the former had a high probability of turnover intention. Job satisfaction was also higher in this group with an OR of 0.75 than in the group that did not experience violence, which had an OR of 0.67. Among individual factors, the group that experienced violence revealed that gender, age, and education all affected turnover intention, while the group that did not experience violence revealed that only age affected turnover intention.

The probability of women’s turnover intention was high in both the groups that did and did not experience violence. In particular, the probability of turnover intention for women in the former group was higher than that of women in the latter group (OR = 1.05 times) at OR = 3.24. Nurses had the highest probability of turnover intention among LTC workers, regardless of whether they had or had not experienced violence. In both groups, a lower position revealed a higher probability of turnover intention. The group that experienced client violence had a higher probability of turnover intention as the position in the facility was lower. Stress factors also affected turnover intention in both groups, with the group that experienced violence having a higher probability of turnover intention due to stress than the group that did not experience violence.

## 4. Discussion

This study analyzed the factors that affect turnover intention among LTC workers using a large-scale survey conducted by the government after the introduction of the Korean LTCI in 2008. LTC workers are the core workforce of LTC services for older adults and may spend much time with them, depending on their health conditions. Therefore, it is a relationship that requires intimacy and trust. However, frequent job changes among care workers can also impose significant stress on older adults receiving LTC services.

We found that 17.8% of LTC workers had turnover intention. With regard to occupations, the highest turnover intention percentages were for social workers (28.2%), nurses (26.6%), and care workers (13.4%). This finding is similar to a recent study that examined job stress among care facility workers, showing that care workers have higher levels of job stress than do nurses. Some local government organizations in Korea also provide a monthly allowance of KRW 20,000 to LTC workers who have worked in a care facility for more than 3 months and provided care services for 60 h or more per month.

The percentage of LTC workers who experienced client violence and reported turnover intention was 35.4%, which is twice the rate of those who reported turnover intention without experiencing violence. Furthermore, the rate of turnover intention among those who experienced violence (24.4%) was higher than that of those who did not experience violence (14.2%), suggesting that experiencing client violence not only leads to personal issues, such as job burnout and turnover intention, but also negatively impacts the quality of care services provided [8,31].

Turnover intention among LTC workers were influenced by various factors, in addition to experiences of violence. Psychological factors, such as job satisfaction and level of satisfaction regarding pay, were found to be lower among those who reported turnover intention. This is consistent with previous research on job satisfaction among social workers [32,33,34,35], nurses [22,24], and care workers [7,15,36]. Factors related to job status, such as being non-regular employees or having a low position, were also found to increase the likelihood of turnover intention [34].

In this study, when comparing turnover intentions between the group two groups, regardless of whether they had experienced violence or not, the group with low job satisfaction and low levels of satisfaction regarding pay reported higher turnover intentions. This suggests that improving working conditions for LTC workers is important to reduce their turnover intention, regardless of whether they have experienced violence or not. However, when looking at specific subgroups, it was found that among those who experienced violence, turnover intention were higher for women and those with higher education levels. This highlights the need for interventions, particularly for women in the LTC workforce, as they are more exposed to violence.

The Korean Ministry of Health and Welfare established four LTC Support Centers (in Seoul, Gyeonggi, Incheon, and Ulsan) in 2020 to protect the rights of LTC workers. However, it is still insufficient for managing the employment of all LTC workers. The more serious issue is that there are no laws or regulations in place to report and punish clients or family members who commit violence in spaces such as homes or facilities. The National Health Insurance Service provides guidelines to clients and families regarding items that cannot be demanded from caregivers. However, this is not a preventive measure against violence. Therefore, it is necessary to consider strong measures such as suspending or restricting the use of LTC services in case of violence.

Recently, in the context of the COVID-19 situation, attempts have been made to provide LTC workers with psychological counseling services through non-face-to-face methods [37]. These attempts have received very positive evaluations for being able to solve the limitations of time and space. Therefore, various means of protecting the rights of LTC workers must be established.

### Limitations

This study could not verify the temporal distribution of violent experiences and the turnover intention through a cross-sectional design. Additionally, due to the use of questions that do not distinguish whether the perpetrator is a client or a client’s family member, the proportion of perpetrators cannot be known and various exposure situations to violence are reduced to simple “yes” or “no” responses. Therefore, in future surveys, specific questions about exposure to violence must be added and additional factors influencing turnover intention should be identified through terminal data collection. Furthermore, follow-up research is needed to determine whether the rate of exposure to violence has decreased following the support provided by national institutions, such as LTC Support Centers.

## 5. Conclusions

This study used the first LTC survey conducted in 2019 by the Korean Ministry of Health and Welfare to identify variables that affect the turnover intention of LTC workers. It revealed that 17.8% of the total respondents were willing to change their jobs. Even more concerning was that a high proportion of them (35.4%) reported experiencing client violence. Moreover, the higher the proportion of individuals who experienced client violence, the higher their turnover intention. The study also revealed a lower age and position, combined with an unstable employment type, resulting in lower job and salary satisfaction and higher turnover intentions. Regarding occupation, nurses’ turnover intention was higher than that of social workers or care workers. This highlights the need to manage stress and prevent client violence in advance in order to reduce the turnover intention of LTC workers. This study can be used as a basic reference for policymakers when formulating policies to not only reduce the turnover intention of LTC workers but also improve the quality of LTC services. Moreover, at the national level, measures should be established to prevent LTC workers from experiencing client violence and help them manage stress; continuous attention is needed to ensure that these requirements are fulfilled.

## Figures and Tables

**Table 1:** Variables included in the logistic regressions with turnover intention as the dependent variable.

Variables	Response Options for Questions
Gender	(0) Male, (1) Female
Age	(1) <29–(6) >70
Education level	(1) Less than high school education, (2) High school, (3) College, (4) University
Turnover intention	(0) No, (1) Yes
Job satisfaction	(1) Not at all satisfied–(5) Completely satisfied
Income satisfaction	(1) Not at all satisfied–(5) Completely satisfied
Type of employment	(0) Non-regular employee, (1) Regular employee
Type of occupation	(1) Nurse, (2) Social worker, (3) Care worker
Type of LTCI benefits	(0) Benefits for home care service, (1) Institutional care benefits
Position	(1) Staff, (2) Assistant manager, (3) Middle manager, (4) Office manager, (5) Facility manager
Experience of client violence	(0) No, (1) Yes
Experience of accidents and illnesses related to work	(0) <0–(3) >3
Stress level	(1) Very low–(5) Very high

**Table 2:** Participant characteristics (*n* = 3541).

Characteristics	Value	Frequency (*n*)	Percentage (%)
Gender	Male	305	8.61
	Female	3236	91.39
Age	<29	114	3.22
	30–39	192	5.42
	40–49	495	13.98
	50–59	1414	39.93
	60–69	1145	32.34
	>70	181	5.11
Education level	Less than high school	764	21.58
	High school	1491	42.11
	College	675	19.06
	University	611	17.26
Violence experienced	No	2289	64.64
	Yes	1252	35.36
Violence type	Verbal and psychological	1050	29.65
	Physical violence	798	22.54
	Sexual violence	400	11.30
Turnover intention	No	2910	82.18
	Yes	631	17.82
Turnover intention from the group without experiences of violence		325	14.20
Turnover intention from the group with experiences of violence		306	24.44

**Table 3:** The proportion of turnover intention based on characteristics of the group with experience of violence (*n* = 3541).

Characteristics	Value	Turnover Intention Rate (%)
Gender	Male	17.02
	Female	25.04
Age	<29	52.0
	30–39	35.06
	40–49	40.47
	50–59	23.07
	60–69	13.09
	>70	2.77
Type of occupation	Nurse	35.74
	Social worker	37.32
	Care worker	17.82

**Table 4:** Participant characteristics with and without turnover intention (*n* = 3541).

Characteristics	Value	Turnover Intention	
		No	Yes
Job satisfaction	Not at all satisfied	15 (60.0)	10 (40.0)
	Quite dissatisfied	138 (58.97)	96 (41.03)
	Average	893 (74.67)	303 (25.33)
	Quite satisfied	1480 (87.89)	204 (12.11)
	Completely satisfied	384 (905.52)	18 (4.48)
Income satisfaction	Not at all satisfied	108 (65.85)	56 (34.15)
	Quite dissatisfied	605 (71.51)	241 (28.49)
	Average	993 (81.86)	220 (18.14)
	Quite satisfied	965 (90.27)	104 (9.73)
	Completely satisfied	239 (95.98)	10 (4.01)
Type of employment	Non-regular employee	1242 (82.25)	268 (17.75)
	Regular employee	1668 (82.13)	363 (17.87)
Type of occupation	Nurse	414 (73.40)	150 (26.60)
	Social worker	398 (71.84)	156 (28.16)
	Care worker	2098 (86.59)	325 (13.41)
Type of LTC benefits	Institutional care benefits	1145 (81.55)	259 (18.45)
	Benefits for home care service	1765 (82.59)	372 (17.41)
Position	Staff	2443 (82.45)	520 (17.55)
	Assistant manager	61 (77.22)	18 (22.78)
	Middle manager	309 (79.43)	80 (20.57)
	Office manager	60 (84.51)	11 (15.49)
	Facility manager	37 (94.87)	2 (5.13)
Experience of accidents and illnesses related to work	0	2327 (83.74)	452 (16.26)
	1	484 (77.32)	142 (22.68)
	2	86 (73.50)	31 (26.50)
	3<	13 (68.42)	6 (31.58)
Stress level	Very low	157 (96.91)	5 (3.09)
	Low	548 (91.03)	54 (8.97)
	Normal	1166 (85.55)	197 (14.45)
	High	783 (75.51)	254 (24.49)
	Very High	256 (67.90)	121 (32.10)

**Table 5:** Factors influencing turnover intention (*n* = 3541).

Variable	β	SE	OR
Job satisfaction (ref. not at all satisfied)	−0.56 ***	0.03	0.57
Income satisfaction (ref. not at all satisfied)	−0.34 ***	0.04	0.70
Type of employment (ref. non-regular employee)	−0.23 *	0.08	0.79
Type of occupation (ref. nurses)			
Social worker	−0.14	0.14	0.86
Care worker	−0.64 ***	0.07	0.52
Type of LTCI benefit (ref. benefits for home care service)	0.07	0.11	1.07
Position (ref. staff)	−0.22 ***	0.53	0.79
Experience of client violence (ref. no)	0.22 *	0.13	1.25
Experience of accidents and illnesses related to work (ref. no)	0.17 *	0.10	1.19
Stress level (ref. very low)	0.32 ***	0.07	1.37
Gender (ref. male)	0.43 *	0.29	1.54
Age	−0.47 ***	0.35	0.62
Education level (ref. less than high school)	0.17 **	0.07	1.19

* *p* < 0.05; ** *p* < 0.01; *** *p* < 0.001.

**Table 6:** Factors affecting turnover intention by experience of violence.

	Group That Had Experienced Client Violence (*n* = 1252)			Group That Had Not Experienced Client Violence (*n* = 2289)		
Variable	β	SE	OR	β	SE	OR
Job satisfaction (ref. not at all satisfied)	−0.53 ***	0.59	0.58	−0.58 ***	0.53	0.55
Income satisfaction (ref. not at all satisfied)	−0.29 ***	0.06	0.74	−0.40 ***	0.52	0.66
Type of employment (ref. non-regular employee)	−0.189	0.13	0.82	−0.24	0.11	0.78
Type of occupation (ref. nurse)						
Social worker	−0.14	0.21	0.86	−0.14	0.19	0.86
Care worker	−0.58 **	0.11	0.55	−0.63 **	0.10	0.53
Type of LTC benefits (ref. benefits for home care service)	−0.04	0.14	0.95	0.12	0.17	1.13
Position (ref. staff)	−0.19 *	0.08	0.82	−0.26 **	0.07	0.76
Stress level (ref. very low)	0.41 ***	0.13	1.50	0.27 ***	0.09	1.31
Gender (ref. male)	1.17 ***	1.07	3.24	0.44	0.24	1.04
Age	−0.47 ***	0.05	0.62	−0.47 ***	0.04	0.62
Education level	0.31 **	0.14	1.36	0.09	0.09	1.09

* *p*< 0.05; ** *p* < 0.01; *** *p* < 0.001.

## Data Availability

Details and downloads of data used in the study can be found at the following address. URL of datasets analyzed: (https://mdis.kostat.go.kr/ofrData/selectOfrDataDetail.do?survId=1004220&itmDiv=2&nPage=3&itemId=2007&itemNm=) (accessed on 11 December 2022).
